# YOLO-Spectra-Net: Reliability-Aware RGB–Infrared Fusion for Robust Object Detection Under Adverse Conditions

**DOI:** 10.3390/s26196028

**Published:** 2026-09-23

**Authors:** Zhangshuo Hu, Lin Chai

**Affiliations:** Sipailou Campus, Southeast University, Nanjing 210096, China; 213232409@seu.edu.cn

**Keywords:** RGB–infrared fusion, multimodal sensing, robust object detection, reliability-aware fusion, infrared imaging, adverse conditions

## Abstract

**Highlights:**

**What are the main findings?**

- A reliability-aware RGB–infrared detection framework is developed for adverse sensing conditions.
- Cross-modal global fusion and conflict-aware spatial suppression reduce error propagation from degraded modalities.

**What are the implications of the main findings?**

- The proposed framework improves robust multimodal detection under challenging sensing conditions.
- Its relatively lightweight design supports practical deployment.

**Abstract:**

Robust object detection in practical sensing systems remains challenging under low visibility, rapidly varying illumination, occlusion, and cluttered backgrounds. Visible images provide rich structural and texture information but are vulnerable to darkness, haze, and glare, whereas infrared images preserve thermal saliency but may suffer from weak spatial detail and thermal crossover. Conventional RGB–infrared fusion strategies often assign fixed or symmetric importance to the two modalities, allowing degraded features to propagate through the detector and impairing perception stability. To address this issue, we propose YOLO-Spectra-Net (SNYOLO), a reliability-aware RGB–infrared fusion framework for adverse-condition object detection. SNYOLO estimates modality reliability and integrates it into adaptive feature fusion, multi-scale perception enhancement, and conflict-aware spatial suppression. The proposed design adaptively emphasizes reliable modality cues, attenuates locally inconsistent responses, and stabilizes multi-scale feature representations while maintaining a relatively lightweight multimodal inference structure. Experiments on a self-collected railway monitoring dataset and two public RGB–infrared benchmarks, M3FD and FLIR, demonstrate improved robustness and stable target-focused responses under challenging sensing conditions. SNYOLO achieves 82.7% mAP@0.5 on M3FD and 79.1% mAP@0.5 on FLIR, with competitive localization accuracy and lower computational cost than most compared multimodal baselines.

## 1. Introduction

Robust object detection under adverse sensing conditions is essential for surveillance, traffic monitoring, railway inspection, and other safety-critical applications. Visible images provide texture, color, and contour information, but their quality can deteriorate sharply under low illumination, haze, smoke, glare, and cluttered backgrounds. Infrared sensing complements visible imaging by capturing thermal radiation that is less dependent on ambient light and can preserve target saliency at night or in low-visibility scenes. However, infrared images usually contain weaker texture and lower spatial detail, and they can also be affected by thermal crossover. Therefore, practical RGB–infrared perception requires not only feature complementarity but also reliability-aware adaptation to changing sensor quality.

Infrared imaging has been widely adopted in night-time, frontlighting, backlighting, and other low-visibility scenarios [1]. RGB–infrared detection has consequently attracted increasing attention in traffic monitoring, security inspection, and autonomous driving. Nevertheless, RGB and infrared data differ substantially in imaging mechanisms, noise distribution [2], spatial texture, and semantic representation. Directly combining the two modalities may introduce redundant or even conflicting responses, particularly when one sensor is locally degraded. Many existing methods still treat both modalities symmetrically or apply fixed fusion rules, which limits their ability to respond to scene-dependent reliability variations.

Existing multimodal detectors generally perform cross-modal interaction through concatenation, weighted summation, or attention-based fusion. Although these strategies improve representation capacity, several limitations remain:

(1) Fixed or symmetric fusion strategies cannot adequately adjust modality contributions when sensing quality changes. For example, IAF R-CNN [3] adopts a dual-branch structure to fuse RGB and infrared features and improves low-illumination detection, but its fusion procedure relies on predefined interactions. TarDAL [4] introduces dual-layer attention to enhance cross-modal representation, yet the overall fusion structure remains relatively symmetric and provides limited explicit modeling of modality reliability.

(2) Many cross-modal interaction mechanisms operate mainly at the channel or global level, while local semantic inconsistency and spatial conflict are insufficiently modeled. DAMSDet [5] enhances feature representation through channel and spatial attention, but its attention formulation does not explicitly suppress locally inconsistent cross-modal responses. CFT [6] uses a Transformer to model long-range cross-modal dependencies, but fine-grained spatial conflict remains difficult to characterize efficiently.

(3) Multi-scale fusion and region-level reliability modeling are often designed as separate components. MSDS-RCNN [7] improves multi-scale detection through dynamic feature selection, but it mainly focuses on scale variation rather than local modality disagreement. YOLO-MS [8] introduces multimodal and multi-scale fusion into a YOLO framework, but region-level reliability and scale-specific fusion remain weakly coupled. Consequently, detector responses may become unstable when input quality varies across modalities and scales.

To address these challenges, we propose YOLO-Spectra-Net (SNYOLO), a practical reliability-aware RGB–infrared fusion framework built on a YOLO-style detector [9]. The framework combines CMGF-based adaptive fusion, multi-scale perception enhancement, and conflict-aware spatial suppression. Within CMGF, spatial–channel attention, cross-modal global affinity learning, and adaptive gating regulate RGB–infrared information flow at feature-pyramid levels, while spatial suppression attenuates local responses caused by RGB–infrared disagreement.

Experiments on a self-collected railway monitoring dataset and two public RGB–infrared benchmarks, M3FD [4] and FLIR [10], demonstrate that SNYOLO improves robustness and maintains stable target-focused responses under challenging sensing conditions. The results indicate that explicit reliability modeling is beneficial for both real-scene deployment and public benchmark evaluation.

The main contributions are summarized as follows:

(1) A reliability-aware multimodal detection framework is proposed to explicitly model modality degradation and incorporate reliability cues into feature-level RGB–infrared fusion.

(2) A Cross-Modal Global Fusion (CMGF) module is designed to model spatial–channel importance, global cross-modal relationships, and adaptive information flow, thereby reducing error propagation from degraded modalities.

(3) A multi-scale perception strategy with conflict-aware spatial suppression is developed to stabilize fused features under scale variation and local RGB–infrared inconsistency.

## 2. Related Work

### 2.1. Multimodal Fusion Methods

Multimodal fusion aims to integrate complementary sensor cues into a unified representation. In RGB–infrared perception, effective fusion must bridge distribution gaps and semantic discrepancies between modalities, which become more pronounced under adverse environmental conditions [1,7]. Early fusion approaches commonly rely on feature concatenation or fixed-weight addition. Although simple, these strategies may introduce redundancy and amplify noise when one modality is degraded.

Recent studies have introduced attention-based fusion to improve feature selectivity and cross-modal interaction [11]. Channel and spatial attention emphasize discriminative responses but may overlook local modality disagreement [12,13]. Cross-modality and multi-scale attention mechanisms further model correlations across modalities and feature levels; however, many designs remain symmetric and can still propagate unreliable responses when modality quality varies dynamically [3,11,14,15].

### 2.2. RGB–Infrared Object Detection

RGB–infrared object detection integrates multimodal fusion into detection pipelines, typically through dual-branch feature extraction and intermediate feature interaction. Classical single-modality detectors, including Faster R-CNN [16], SSD [17], and dense one-stage variants based on focal loss [18], provide strong detection foundations but do not explicitly address cross-modal discrepancies. The development of deep convolutional architectures, including influential multi-branch designs such as GoogLeNet [19], has also established important foundations for hierarchical and multi-scale visual feature extraction. Representative multispectral detectors such as IAF R-CNN [3], TarDAL [4], CFT [6], and CMAFF [15] demonstrate that multimodal cues can improve robustness over single-modality baselines. Nevertheless, detection performance may fluctuate when the relative reliability of RGB and infrared inputs changes across scenes, motivating fusion strategies that explicitly encode modality reliability.

### 2.3. Robustness and Reliability Modeling

Robust multimodal perception requires explicit handling of modality degradation because the reliability of each sensor may vary across inputs and even across spatial regions. Without reliability modeling, fusion modules can propagate errors from degraded modalities into the fused representation and reduce detection stability. Recent studies have attempted to reduce cross-modality redundancy, adapt feature selection, and align attention responses under adverse conditions [1,5,15]. However, many methods estimate reliability only at the global or channel level and do not explicitly convert local RGB–infrared disagreement into a spatial suppression signal. Reliability estimation and suppression of unreliable responses are therefore central to stabilizing fused features in practical sensing scenarios.

Table 1 summarizes representative single-modality detectors used as detection foundations, and Table 2 summarizes attention mechanisms commonly adopted for multimodal fusion. These comparisons motivate a framework that jointly considers CMGF-based adaptive fusion, scale-aware perception, and local conflict suppression rather than relying on a single attention formulation.

## 3. Method

### 3.1. Overall Network Architecture

This work presents YOLO-Spectra-Net (SNYOLO), a reliability-aware RGB–infrared fusion framework for robust object detection in challenging sensing environments. The framework accepts aligned RGB–IR image pairs and extracts multi-level features from both modalities through a shared YOLO-style backbone. The paired RGB–IR setting is used consistently for the RailID, M3FD, and FLIR experiments, while single-modality results are included as baselines where appropriate. By embedding reliability estimation into fusion and feature enhancement, SNYOLO aims to reduce response instability caused by illumination variation, sensor noise, low visibility, and cross-modal inconsistency while maintaining efficient inference.

The framework is organized around three mechanisms for adverse-condition sensing:

(1) Modality reliability estimation to reflect scene-dependent sensor quality,

(2) Adaptive cross-modal fusion with conflict suppression to reduce unstable fusion when one modality is degraded,

(3) Multi-scale consistency modeling to maintain stable detection across target sizes.

At the network level, reliability-aware fusion is integrated into a standard detection pipeline composed of four main stages, with RMPF and CASS embedded in the neck and detection head, respectively:

(1) Dual-stream shared YOLO backbone network (Backbone) [8],

(2) Multi-scale Cross-Modal Global Fusion (CMGF) module,

(3) Cross-scale feature aggregation neck network with the RMPF module (YOLO Neck/PAN) [20],

(4) Multi-scale detection heads preceded by the CASS module (Detect Head).

Given aligned RGB and infrared input images,

(1) $$I^{rgb},I^{ir}\in R^{3\times 640\times 640},$$

SNYOLO uses a YOLO-style dual-stream backbone with shared weights to extract features from the two modalities, as illustrated in the overall framework in Figure 1. The detailed arrangement of the Backbone, CMGF, RMPF, CASS, Neck, and detection heads is shown in Figure 2. The two branches share the same topology and parameters, which constrains RGB and infrared features into a unified representation space while reducing the parameter count. This shared-weight design also helps alleviate modality distribution gaps and improves cross-modal consistency and generalization.

The backbone network is based on the C2f/CSP structure and outputs multi-layer features at three scales:

(2) $$F_{l}^{m}=B\left(I^{m}\right),\;m\in \left\{rgb,ir\right\},\;l\in \left\{P3,P4,P5\right\},$$

where B(·) denotes the shared YOLO backbone, $F_{l}^{m}\in R^{C_{l}\times H_{l}\times W_{l}}$ is the feature map of modality *m* at scale *l*, and the channel numbers of the three layers are:

(3) $$C_{P3}=40,C_{P4}=72,C_{P5}=136.$$

To exploit RGB and infrared information at different receptive fields, a Cross-Modal Global Fusion (CMGF) module is applied at each scale. For each scale *l*, CMGF receives $F_{l}^{rgb}$ and $F_{l}^{ir}$, models cross-modal relationships and adaptive gating responses, and generates the fused representation:

(4) $$F_{l}^{cmgf}=CMGF\left(F_{l}^{rgb},F_{l}^{ir}\right).$$

This design allows cross-modal contributions to vary with spatial context and feature relationships, which is important under weak illumination, occlusion, and other degraded conditions.

The fused multi-scale features are then passed to the YOLO neck with path aggregation (PAN) for cross-scale feature aggregation. Within the neck, RMPF is applied along the multi-scale aggregation paths to combine local and contextual cues while preserving spatial structure. The top-down and bottom-up paths exchange semantic and spatial information across feature levels, improving detection for objects of different sizes:

(5) $$\left\{F_{P3}^{neck},F_{P4}^{neck},F_{P5}^{neck}\right\}=N\left(F_{P3}^{cmgf},F_{P4}^{cmgf},F_{P5}^{cmgf}\right),$$

where N(·) denotes the complete PAN-based neck with the embedded RMPF enhancement. Its three inputs are the CMGF-P3, CMGF-P4, and CMGF-P5 features. In the top-down path, the P5 feature is progressively upsampled and combined with the corresponding P4 and P3 features through lateral connections and feature concatenation. RMPF is applied along the multi-scale aggregation paths to enhance local and contextual representations. The subsequent bottom-up path re-aggregates the enhanced features across scales, producing Neck-P3, Neck-P4, and Neck-P5, which are forwarded to the corresponding detection heads. Therefore, CMGF-P3 is explicitly used as the shallow-scale input of the PAN neck, as shown in the detailed network structure in Figure 2.

Before the corresponding detection heads, CASS uses the RGB–IR feature difference to suppress spatially conflicting responses in the fused features. The resulting features at different scales are then forwarded to the corresponding detection heads:

(6) $$F_{l}^{cass}=CASS\left(F_{l}^{neck},F_{l}^{rgb},F_{l}^{ir}\right),\;F_{P3}^{cass}\to {\mathrm{Detect}}_{P3},\;F_{P4}^{cass}\to {\mathrm{Detect}}_{P4},\;F_{P5}^{cass}\to {\mathrm{Detect}}_{P5},$$

where l∈{P3,P4,P5}. These heads are responsible for small, medium, and large objects and predict class probabilities, bounding-box locations, and confidence scores.

Overall, the shared backbone provides a unified feature space, while adaptive cross-modal fusion and cross-scale aggregation stabilize representations under sensing degradation and scale variation. The main components are described below.

### 3.2. Cross-Modal Global Fusion (CMGF) Module

RGB and infrared features contain complementary information but also exhibit modality-specific noise, semantic discrepancies, and local response inconsistency. Direct concatenation or element-wise addition cannot explicitly model the correspondence between spatial locations in the two modalities and may propagate unreliable responses into the fused representation. To address this issue, SNYOLO introduces the Cross-Modal Global Fusion (CMGF) module, which combines feature projection, dual-path spatial–channel attention, cross-modal global affinity learning, and adaptive gating. These operations are related to established spatial–channel attention, non-local interaction, and multimodal gating designs [12,13,14,21]. The complete CMGF structure is illustrated in Figure 3.

CMGF is inserted at each feature-pyramid level, namely P3, P4, and P5. For clarity, the operation at one feature level is described below. Let the RGB and infrared features produced by the shared dual-stream backbone be:

(7) $$F^{rgb},F^{ir}\in R^{C\times H\times W},$$

where *C*, *H*, and *W* denote the channel number, height, and width, respectively. First, a 1×1 convolution followed by batch normalization projects the two modalities into a common semantic space while retaining the original features through a residual connection:

(8) $${\tilde{F}}^{m}=\mathrm{BN}({\mathrm{Conv}}_{1\times 1}\left(F^{m}\right))+F^{m},\;m\in \left\{rgb,ir\right\}.$$

This residual projection preserves an identity path for the original modality features, following the general residual-learning principle of facilitating stable feature propagation [22]. It also reduces the representation discrepancy between the two modalities and provides a shared feature space for subsequent cross-modal interaction.

#### 3.2.1. Dual-Path Spatial–Channel Attention

CMGF uses spatial and channel attention in parallel to describe where informative responses occur and which channels carry discriminative semantics, following the complementary attention principle used in established attention modules [12,13]. For spatial attention, channel-wise global average pooling and global max pooling are applied to the projected feature:

(9) $$S^{m}=\sigma \left({\mathrm{Conv}}_{7\times 7}\left(\left[{\mathrm{GAP}}_{c}\left({\tilde{F}}^{m}\right);{\mathrm{GMP}}_{c}\left({\tilde{F}}^{m}\right)\right]\right)\right),$$

where ${\mathrm{GAP}}_{c}$ and ${\mathrm{GMP}}_{c}$ aggregate the channel dimension, σ(·) denotes the Sigmoid function, and $S^{m}\in R^{1\times H\times W}$ is the spatial attention map. The average-pooling branch describes the overall response distribution, whereas the max-pooling branch preserves salient local responses.

Channel attention is calculated from spatially pooled descriptors:

(10) $$C^{m}=\sigma \left(\mathrm{MLP}\left({\mathrm{GAP}}_{s}\left({\tilde{F}}^{m}\right)\right)+\mathrm{MLP}\left({\mathrm{GMP}}_{s}\left({\tilde{F}}^{m}\right)\right)\right),$$

where ${\mathrm{GAP}}_{s}$ and ${\mathrm{GMP}}_{s}$ aggregate the spatial dimensions and $C^{m}\in R^{C\times 1\times 1}$ is the channel attention vector. The MLP uses a bottleneck structure:

(11) $$\mathrm{MLP}\left(x\right)=W_{2}\mathrm{ReLU}\left(W_{1}x\right),$$

where the intermediate dimension is reduced by a factor of *r* to control the additional parameters. The spatial and channel branches are applied element-wise in the subsequent fusion stage.

#### 3.2.2. Cross-Modal Global Affinity Learning

To capture long-range correspondence between RGB and infrared responses, the projected features are flattened into spatial tokens. This formulation follows the general idea of non-local feature interaction, while the affinity is computed here across the RGB and infrared modalities [14,15]:

(12) $$Q^{m}=\mathrm{Flatten}\left({\tilde{F}}^{m}\right)\in R^{HW\times C}.$$

For each modality and its complementary modality $\bar{m}$, a scaled dot-product affinity matrix is computed:

(13) $$A^{m\to \bar{m}}=\mathrm{Softmax}\left(\frac{Q^{m}{\left(Q^{\bar{m}}\right)}^{T}}{\sqrt{C}}\right)\in R^{HW\times HW}.$$

The matrix $A^{m\to \bar{m}}$ measures the correspondence between all spatial locations of the two modalities. The complementary feature aligned to modality *m* is then obtained by affinity-weighted aggregation:

(14) $$F^{\bar{m}\to m}=\mathrm{Reshape}\left(A^{m\to \bar{m}}Q^{\bar{m}}\right)\in R^{C\times H\times W}.$$

Unlike local concatenation, this operation allows each location in one modality to retrieve semantically related information from the complete feature map of the other modality.

#### 3.2.3. Adaptive Gated Fusion

The original and cross-modal features are combined through a learned spatial gate. Adaptive gating is widely used to regulate the contribution of complementary information in multimodal fusion [21]. For modality *m*, the gate is generated from the projected feature and its cross-modal counterpart:

(15) $$\alpha^{m}=\sigma \left({\mathrm{Conv}}_{1\times 1}\left[{\tilde{F}}^{m};F^{\bar{m}\to m}\right]\right),$$

where $\alpha^{m}\in R^{1\times H\times W}$ controls the contribution of cross-modal information at each spatial location. The modality-specific output is defined as:

(16) $$F_{G}^{m}=F^{m}+S^{m}⊙C^{m}⊙\left(\alpha^{m}⊙F^{\bar{m}\to m}+\left(1-\alpha^{m}\right)⊙{\tilde{F}}^{m}\right),\;m\in \left\{rgb,ir\right\},$$

where ⊙ denotes element-wise multiplication. The residual term preserves modality-specific low-level information, while the attention maps and adaptive gate regulate the strength of spatially aligned and semantically complementary features.

Finally, the two modality-specific outputs are concatenated and projected to produce the single fused feature required by the detection neck:

(17) $$F^{cmgf}={\mathrm{Conv}}_{1\times 1}\left[F_{G}^{rgb};F_{G}^{ir}\right]\in R^{C\times H\times W}.$$

The same CMGF formulation is applied independently at P3, P4, and P5. Parameters are not shared across pyramid levels so that the module can adapt its cross-modal correspondence and gating behavior to the different receptive fields of small, medium, and large targets. By jointly modeling global cross-modal dependencies and local spatial–channel importance, CMGF preserves complementary modality information while limiting the propagation of modality-specific noise before multi-scale feature aggregation.

### 3.3. Region-Aware Multi-Scale Perception Fusion (RMPF)

Targets in adverse scenes vary substantially in scale, shape, and spatial distribution. A single receptive field is often insufficient to capture both fine local details and broader contextual information, which may lead to missed small targets or incomplete large-object representation [20]. Such limitations become more pronounced when visibility is poor or background interference is strong.

Scale variation can also amplify cross-modal misalignment across the feature pyramid, resulting in unstable responses at different detection levels. Multi-scale consistency modeling is therefore introduced to aggregate multi-receptive-field cues and stabilize fused features under large scale changes.

Let the input feature be:

(18) $$F\in R^{C\times H\times W},$$

which is obtained from the backbone output or the preceding fusion module.

To introduce multi-receptive-field information at the same feature level, RMPF constructs parallel convolutional branches for the input feature. Because modality responses may vary across scales, three convolutional kernels, 3×3, 5×5, and 7×7, are used:

(19) $$F_{1}={\mathrm{Conv}}_{3\times 3}\left(F\right),\;F_{2}={\mathrm{Conv}}_{5\times 5}\left(F\right),\;F_{3}={\mathrm{Conv}}_{7\times 7}\left(F\right),$$

where all branches preserve the spatial size H×W and differ only in receptive field. Smaller kernels retain local texture and edge details, whereas larger kernels capture broader contextual cues. This parallel design enriches scale information without changing feature resolution.

The obtained multi-scale features are concatenated in the channel dimension:

(20) $$F^{ms}=\mathrm{Concat}\left(F_{1},F_{2},F_{3}\right),\;F^{ms}\in R^{(C_{1}+C_{2}+C_{3})\times H\times W},$$

where features from different receptive fields are merged into a common feature space. This design avoids additional downsampling or scale transformation, allowing multi-scale context to be used while preserving the original spatial structure.

RMPF further models spatial importance. Since targets typically occupy only part of an image, background regions may introduce substantial noise. Multi-scale features alone cannot always distinguish informative target regions from redundant responses. Therefore, RMPF introduces a region-weighting mechanism in which a 1×1 convolution compresses channels and a Sigmoid function produces a spatial weight map:

(21) $$M=\sigma \left({\mathrm{Conv}}_{1\times 1}\left(F^{ms}\right)\right),\;M\in R^{1\times H\times W},$$

where M represents the learned importance weight of each spatial location. Informative regions are assigned higher weights, whereas background responses are suppressed.

The spatial weights are then applied to the multi-scale features, as illustrated in Figure 4:

(22) $$F^{w}=F^{ms}⊙M,$$

where ⊙ denotes element-wise multiplication. This operation reweights spatial responses and encourages the model to focus on likely target regions rather than irrelevant background.

Direct weighting may attenuate useful contextual information. To mitigate this risk, a residual refinement branch is added at the output. The weighted features are first processed by a 3×3 convolution and ReLU activation:

(23) $$F^{r}=\mathrm{ReLU}\left({\mathrm{Conv}}_{3\times 3}\left(F^{w}\right)\right),$$

and then added back to the weighted features:

(24) $$F^{out}=F^{w}+F^{r}.$$

The residual branch introduces nonlinearity while preserving the original multi-scale regional cues, reducing feature weakening and improving response stability.

By combining parallel multi-scale modeling with region weighting, RMPF preserves local detail while incorporating broader context at each spatial location. Compared with single-scale representations, this design better adapts to target-size variation and complex layouts, providing stronger features for detection.

### 3.4. Conflict-Aware Spatial Suppression Module (CASS)

In multimodal fusion, RGB and infrared features may respond differently at the same spatial location because of noise, occlusion, thermal ambiguity, or local sensor degradation. Preserving these inconsistent responses can amplify interference and degrade detection accuracy [3]. Unstable local activations may then propagate through subsequent layers and reduce robustness under adverse conditions.

Strong cross-modal disagreement indicates that the sensing cues at specific locations are unreliable. Suppressing such responses during feature refinement can reduce ambiguous activation propagation and stabilize the fused feature distribution.

From a feature representation perspective, modality inconsistency acts as a source of spatial interference. Larger response gaps imply stronger local disagreement and lower semantic reliability. Suppressing these high-conflict regions reduces interference propagation, improves spatial response consistency, and contributes to stable perception under environmental variation.

Based on this observation, we design the Conflict-Aware Spatial Suppression module (CASS) [15]. CASS screens fused features in the spatial dimension by explicitly modeling inter-modality differences, thereby suppressing ambiguous activations caused by RGB–infrared disagreement. Let the features from the infrared and visible branches be:

(25) $$F^{ir},F^{rgb}\in R^{C\times H\times W},$$

and let the current fused feature be:

(26) $$F^{fused}\in R^{C\times H\times W}.$$

To characterize spatial modality differences, the absolute element-wise difference between the two modality features is computed:

(27) $$F^{conf}=\left|F^{ir}-F^{rgb}\right|.$$

This operation directly measures local response gaps between modalities. Larger values indicate stronger inconsistency and usually correspond to noise, ambiguity, or unreliable cues.

Because cross-modal conflict mainly appears as a spatial distribution difference, the conflict feature is compressed along the channel dimension to obtain a single-channel representation:

(28) $$F_{s}^{conf}=\frac{1}{C}\sum_{c=1}^{C}F_{c}^{conf},\;F_{s}^{conf}\in R^{1\times H\times W},$$

where *c* indexes the feature channels and *s* denotes the spatially aggregated conflict representation. This preserves spatial disagreement information while reducing subsequent computation.

Spatial conflict weights are then generated through a 1×1 convolution followed by Sigmoid activation:

(29) $$M^{conf}=\sigma \left({\mathrm{Conv}}_{1\times 1}\left(F_{s}^{conf}\right)\right).$$

The resulting weight map indicates conflict strength at each location within the range [0,1]. Larger values correspond to stronger modality differences and higher uncertainty.

To transform conflict information into a suppression effect, reverse mapping is applied:

(30) $$M^{sup}=1-M^{conf}.$$

Thus, consistent regions retain larger weights, whereas high-conflict locations receive smaller weights, forming a spatial suppression mechanism.

Finally, the suppression weights are applied to the fused feature:

(31) $$F^{cass}=F^{fused}⊙M^{sup},$$

where ⊙ represents element-wise multiplication. This operation reweights spatial responses without changing the feature size or channel number, attenuating high-conflict regions while preserving more consistent regions.

Through this process, illustrated in Figure 5, interference in the fused feature is suppressed and responses are concentrated in more reliable regions. Compared with direct fusion, CASS reduces uncertainty caused by modality differences, stabilizes the feature distribution, and provides more reliable inputs for detection.

Together, CMGF, RMPF, and CASS address different sources of instability, including modality degradation, scale-specific fusion imbalance, multi-scale response inconsistency, and local cross-modal conflict. CMGF performs adaptive cross-modal interaction through spatial–channel attention, global affinity learning, and learned gating, whereas CASS explicitly suppresses local responses with strong RGB–IR disagreement.

Unlike conventional spatial attention, CASS uses the absolute RGB–IR feature difference as the input to the suppression map. The generated mask is therefore associated with cross-modal disagreement rather than activation magnitude alone. This design implements spatial reliability modeling directly: regions with high disagreement are treated as uncertain and down-weighted before detection.

## 4. Experiments and Results Analysis

### 4.1. Datasets

To evaluate robustness under modality degradation and assess generalization across different sensing scenarios, we use a self-collected dataset and two public RGB–infrared benchmarks. These datasets cover diverse illumination conditions, environmental variations, and background complexity, enabling systematic analysis of fusion stability under adverse scenarios and fair comparison with representative methods.

(1) RailID-Dataset (Self-collected Railway Train Identification Dataset)

We construct a self-collected train identification dataset, termed RailID-Dataset. The images are collected from real-world surveillance scenes and include diverse viewpoints, motion blur, cluttered backgrounds, and strong illumination variation such as front-light and back-light conditions. These factors commonly degrade visual features and induce unstable detector responses, making the dataset suitable for evaluating robustness under complex interference.

The dataset is manually annotated and cleaned, and only samples with clear target regions are retained. It contains 500 aligned RGB–IR pairs, including 400 pairs for training and 100 pairs for testing. Therefore, RailID-Dataset supports RGB-only, infrared-only, and paired RGB + IR evaluations, including multimodal fusion and ablation studies.

Low-visibility weather, such as rain and fog, can significantly degrade RGB image quality and destabilize feature extraction. Because collecting large-scale paired low-visibility samples is costly, synthetic fog is applied to the RGB image in each RailID-Dataset pair while the corresponding infrared image is retained. This preserves the paired structure and increases environmental diversity for evaluating multimodal robustness. The generation process follows the classical atmospheric scattering model and dark channel prior theory [23]. Given a clear image J(x), the foggy image I(x) is generated as

(32) I(x)=J(x)·t(x)+A·(1−t(x)),

where *x* denotes the pixel position, $A\in R^{3}$ is the global atmospheric light intensity vector, and t(x)∈[0,1] is the transmittance.

The transmittance t(x) is estimated using the dark channel prior. The dark channel is defined as

(33) $$J^{dark}\left(x\right)=\min_{y\in \Omega (x)}\left(\min_{c\in \{r,g,b\}}J^{c}\left(y\right)\right),$$

where Ω(x) denotes a local window centered at *x*, and $J^{c}$ is the *c*-th color channel.

The transmittance is estimated as

(34) $$t\left(x\right)=1-\omega \;\cdot \;J^{dark}\left(x\right),$$

where ω∈(0,1] is the fog concentration adjustment coefficient.

To avoid numerical instability caused by excessively low transmittance, a lower-bound constraint is applied:

(35) $$t\left(x\right)=\max(t\left(x\right),t_{0}),$$

where $t_{0}$ is the minimum transmittance threshold.

Synthetic fog images are generated using the above model and used to construct training samples under low-visibility conditions.

By controlling transmittance attenuation and atmospheric light, synthetic samples with different fog densities are generated. The fogged images preserve the structural and semantic content of the original scenes while reducing contrast and detail visibility, making the dataset more representative of complex adverse conditions.

(2) M3FD Dataset

To evaluate performance on general multimodal detection tasks, we use M3FD (Multi-modal Multi-spectral Fusion Dataset). M3FD provides paired visible and infrared images across diverse scenes, multiple object categories, and complex backgrounds. It is widely used for evaluating multimodal fusion and detection. Experiments on M3FD assess the generalization ability of the proposed method beyond railway scenarios.

(3) FLIR Dataset

The FLIR multispectral dataset is also used for comparison. FLIR contains real road and monitoring scenes, provides high-quality visible and thermal images, and includes standard annotations. It is a common benchmark for multispectral detection and supports comparison with existing methods under realistic sensing conditions.

Overall, RailID-Dataset is used to analyze RGB–infrared fusion robustness under complex interference and low visibility in a controlled railway setting, while M3FD and FLIR are used to evaluate generalization on public paired benchmarks.

### 4.2. Experimental Environment and Implementation Details

All experiments are conducted under the same hardware and software settings to ensure fair and consistent comparisons. Training and testing are implemented in PyTorch, and all methods follow the same training protocol and key parameter settings.

#### 4.2.1. Computational Platform

The experimental platform is configured as shown in Table 3.

This environment supports efficient training and inference for multimodal detectors with multi-scale fusion and attention-related modules.

#### 4.2.2. Model Implementation Details

SNYOLO is implemented based on the YOLOv12n detection framework. For RailID-Dataset, M3FD, and FLIR, RGB and infrared images are processed by separate feature-extraction branches to generate multi-level features. CMGF, RMPF, and CASS are then integrated into the feature pyramid to enable adaptive cross-modal interaction, multi-scale feature enhancement, and conflict-aware suppression. Single-modality baselines are obtained by disabling the corresponding modality branch and cross-modal fusion when required. Apart from the proposed modules, the remaining network components follow YOLOv12n to avoid unnecessary structural differences and keep the comparison focused on the fusion mechanisms.

All added modules use compact convolutions. Specifically, 1×1 convolutions are used for channel mapping and weight generation, while 3×3 convolutions are used for local feature modeling. Batch normalization and nonlinear activations are adopted at key positions to improve training stability [24]. Adaptive optimizers such as Adam are also widely used for neural-network training [25]; however, stochastic gradient descent (SGD) is used as the optimizer in all experiments in this study.

#### 4.2.3. Training Strategy and Parameter Settings

Model training is performed end-to-end. The loss follows the standard YOLOv12n weighted combination of classification loss, localization loss, and confidence loss. Stochastic gradient descent (SGD) is used as the optimizer. The main training hyperparameters are summarized in Table 4.

Input images are uniformly resized during training, and basic data augmentation strategies, including random flipping, scale transformation, and Mosaic augmentation, are applied. Mosaic augmentation is disabled during the last 10 epochs to promote stable convergence. For the self-constructed RailID-Dataset, original clear RGB–IR pairs and synthetic fog-degraded RGB images together with their corresponding infrared images are jointly used during training to improve robustness under low-visibility conditions. For M3FD and FLIR, the official training and testing splits are followed, and no additional artificial fog augmentation is introduced.

All models are trained for 150 epochs under the same settings, and the final detection results are reported on the test set.

### 4.3. Evaluation Metrics

To evaluate detection performance, model complexity, inference efficiency, and robustness, we use Precision, Recall [26], AP, mAP@0.5, mAP@0.5:0.95 [27], parameter count (Params), floating-point operations (FLOPs), average inference speed in frames per second (average FPS), and Grad-CAM visualization analysis.

(1) Precision

Precision measures the proportion of positive predictions that are correct:

(36) $$\mathrm{Precision}=\frac{TP}{TP+FP},$$

where TP is the number of correct detections and FP is the number of false detections.

(2) Recall

Recall measures the proportion of ground-truth targets that are detected:

(37) $$\mathrm{Recall}=\frac{TP}{TP+FN},$$

where FN is the number of missed targets.

(3) Average Precision (AP)

Average Precision (AP) is defined as the area under the Precision–Recall curve:

(38) $$AP=\int_{0}^{1}P\left(R\right)\;dR,$$

where P(R) represents Precision at different Recall levels.

In practice, AP is computed by numerical integration over the discrete Precision–Recall curve.

(4) mAP@0.5

mAP@0.5 averages AP across categories at an IoU threshold of 0.5:

(39) $$\mathrm{mAP}\text{@}0.5=\frac{1}{N}\sum_{i=1}^{N}{\mathrm{AP}}_{i}^{\mathrm{IoU}=0.5},$$

where *N* is the number of categories and ${\mathrm{AP}}_{i}^{\mathrm{IoU}=0.5}$ is the AP of category *i* at IoU = 0.5.

(5) mAP@0.5:0.95

mAP@0.5:0.95 averages AP across multiple IoU thresholds:

(40) $$\mathrm{mAP}\text{@}0.5\text{:}0.95=\frac{1}{K}\sum_{k=1}^{K}\left(\frac{1}{N}\sum_{i=1}^{N}{\mathrm{AP}}_{i}^{{\mathrm{IoU}}_{k}}\right),$$

where

(41) $${\mathrm{IoU}}_{k}\in \left\{0.5,0.55,0.60,\ldots ,0.95\right\},\;K=10,$$

and *N* is the number of categories. This metric imposes stricter localization requirements and better reflects performance across different overlap levels.

(6) Parameter Count (Params)

Parameter count measures model complexity and is defined as the total number of learnable parameters:

(42) $$\mathrm{Params}=\sum_{l=1}^{L}\left|\Theta_{l}\right|,$$

where $\Theta_{l}$ is the parameter set of layer *l* and *L* is the number of layers. Params are reported in millions (M).

(7) Floating-Point Operations (GFLOPs)

GFLOPs measure the computational cost during inference in billions of floating-point operations:

(43) $$\mathrm{GFLOPs}=\frac{1}{10^{9}}\sum_{l=1}^{L}{\mathrm{FLOPs}}_{l},$$

where ${\mathrm{FLOPs}}_{l}$ is the raw operation count of layer *l*. GFLOPs are calculated using a fixed 640×640 input to ensure comparable complexity analysis.

(8) Average Inference Speed (FPS)

Average inference speed is measured in frames per second (FPS), which indicates the average number of images processed by the detector per second over the complete test set:

(44) $$\mathrm{Average}\;\mathrm{FPS}=\frac{N}{T},$$

where *N* is the number of test images and *T* is the total inference time in seconds. Average FPS is measured with a 640×640 input and batch size one after a fixed warm-up period. All compared models were evaluated on the same NVIDIA RTX 4090 platform under identical software settings, and the reported value was calculated over the same test split. The same timing convention was used for all models.

(9) Pointing Game (PG)

The Pointing Game evaluates whether the most activated location in a class-specific heatmap falls inside the corresponding ground-truth target region [28]. For the *n*-th evaluated heatmap, let $B_{n}$ denote the union of the ground-truth bounding boxes for the target category, and let $\Omega_{n}$ denote all spatial locations in the heatmap. The location of the maximum response is

(45) $$p_{n}^{*}=\underset{\left(i,j\right)\in \Omega_{n}}{\arg\max}\;{\tilde{L}}_{n,ij}^{c},$$

where ${\tilde{L}}_{n}^{c}$ is the non-negative class-specific heatmap. The hit indicator is defined as

(46) $$h_{n}=\left\{\begin{array}{cc} 1, & p_{n}^{*}\in B_{n},\\ 0, & p_{n}^{*}\notin B_{n}. \end{array}\right.$$

The dataset-level Pointing Game score is then calculated as

(47) $$\mathrm{PG}=\frac{1}{N}\sum_{n=1}^{N}h_{n}\times 100\%,$$

where *N* is the number of evaluated heatmaps. A higher PG indicates that the most salient response is more consistently located inside the target region.

(10) Energy-Based Pointing Game (EBPG)

The Energy-Based Pointing Game extends the Pointing Game by measuring the proportion of total heatmap energy contained within the ground-truth target region [29]. For a non-negative heatmap, the EBPG score for the *n*-th sample is defined as

(48) $${\mathrm{EBPG}}_{n}=\frac{\sum_{\left(i,j\right)\in B_{n}}{\tilde{L}}_{n,ij}^{c}}{\sum_{\left(i,j\right)\in \Omega_{n}}{\tilde{L}}_{n,ij}^{c}},$$

and the dataset-level score is

(49) $$\mathrm{EBPG}=\frac{1}{N}\sum_{n=1}^{N}{\mathrm{EBPG}}_{n}\times 100\%.$$

The heatmap is normalized to a non-negative response before calculation, and $B_{n}$ is the union of all ground-truth boxes associated with the evaluated target. EBPG therefore reflects not only whether the strongest point is correctly located, but also whether the overall response energy is concentrated within the target region. Higher EBPG values indicate more target-focused heatmaps.

(11) Grad-CAM Visualization Analysis

In addition to quantitative metrics, Grad-CAM [30] is used to visualize feature responses. It generates heatmaps that indicate class-discriminative regions based on gradient information from target categories.

Let the classification score of category *c* be $y^{c}$, and let the selected feature map of a convolutional layer be:

(50) $$A^{k}\in R^{H\times W},$$

where *k* is the channel index. Grad-CAM first computes the gradients of the class score with respect to the feature maps and then obtains channel weights through global average pooling:

(51) $$\alpha_{k}^{c}=\frac{1}{Z}\sum_{i}\sum_{j}\frac{\partial y^{c}}{\partial A_{ij}^{k}},$$

where Z=H×W and $\alpha_{k}^{c}$ is the weight of channel *k* for class *c*.

The weights are linearly combined with the feature maps and passed through ReLU to obtain the final response map:

(52) $$L_{\mathrm{Grad}\text{-}\mathrm{CAM}}^{c}=\mathrm{ReLU}\left(\sum_{k}\alpha_{k}^{c}A^{k}\right).$$

The heatmap $L_{\mathrm{Grad}-\mathrm{CAM}}^{c}$ indicates the spatial regions that contribute most to the prediction. Upsampling and overlaying it on the original image visually highlights discriminative regions.

In multimodal detection, Grad-CAM heatmaps help compare how different models respond to targets and backgrounds. Concentrated responses around target boundaries suggest effective feature discrimination, whereas scattered or shifted responses indicate residual interference or unstable behavior. Grad-CAM therefore provides qualitative evidence for evaluating the proposed fusion strategy.

### 4.4. Performance Under Challenging Conditions

Robustness is analyzed under conditions that commonly degrade sensing quality, including low illumination, occlusion, haze-like interference, and background clutter. The self-collected paired dataset with synthetic fog augmentation provides a controlled setting for evaluating low-visibility degradation and cross-modal robustness, whereas M3FD and FLIR contain diverse scenes with illumination variation and modality inconsistency. Quantitative comparisons and Grad-CAM analysis are reported to examine whether the proposed fusion strategy suppresses interference and maintains stable target responses under degraded sensing.

To reflect recent progress, the comparison includes classical RGB–IR detectors and newer baselines such as DAMSDet [5] and YOLOv12 [9]. All compared methods are evaluated under matched input resolution and dataset splits whenever possible, and image-fusion baselines are followed by the same detector training protocol to ensure fair detection comparison.

### 4.5. Contribution of Infrared Modality

The contribution of infrared sensing is evaluated by comparing RGB-only, infrared-only, and paired RGB + IR settings on the paired RailID, M3FD, and FLIR datasets. Single-modality baselines quantify the limitations of each sensor under adverse conditions, while paired RGB + IR results demonstrate how complementary thermal cues improve robustness when visible contrast is weak. These comparisons provide direct evidence for the value of infrared sensing under low illumination and background interference. The ablation study is conducted on RailID-Dataset to evaluate the fusion modules on paired RGB–IR data in the railway monitoring scenario.

### 4.6. Practical Applicability Discussion

The proposed framework targets practical requirements including stable detection under challenging visibility, limited computational budget, and robustness to sensor degradation. These properties are relevant to deployments in surveillance, traffic monitoring, railway inspection, and safety-critical monitoring, where illumination changes and partial occlusion are common. The relatively lightweight multimodal design improves robustness without substantially increasing model complexity. Practical value is reflected by improved convergence stability, consistent gains under adverse conditions, and clearer target-focused responses in Grad-CAM visualizations.

### 4.7. Comparison Experiments on Self-Collected Dataset

To evaluate robustness under complex interference and low visibility in a controlled railway setting, YOLO-Spectra-Net is compared with mainstream detectors on the self-constructed RailID-Dataset. SNYOLO uses the original aligned RGB–IR pairs as dual-modal inputs, whereas the other compared single-input detectors use fused images generated by combining the corresponding RGB and infrared images. Thus, the comparison follows the input requirements of each detector while using the same underlying RGB–IR samples. The results in Table 5 are reported with the corresponding input formats, and training convergence curves are shown in Figure 6; Grad-CAM visualizations are presented in Figure 7.

As shown in Table 5, YOLO-Spectra-Net achieves 97.3% mAP@0.5 and 68.2% mAP@0.5:0.95, outperforming the compared methods on this dataset. Compared with lightweight YOLO baselines, YOLOv12n reaches 86.8% mAP@0.5 but shows a lower result on the stricter mAP@0.5:0.95 metric, whereas the proposed method improves both detection confidence and localization quality. The improvement in mAP@0.5:0.95 indicates more consistent boundary regression under complex interference and reflects more stable feature responses.

Precision and Recall further reflect detection robustness. YOLO-Spectra-Net obtains 96.0% Precision and 91.0% Recall, maintaining high detection sensitivity while reducing false alarms. In contrast, Faster R-CNN and CenterNet obtain lower Precision and Recall, indicating that conventional pipelines are less stable for small targets under cluttered backgrounds and strong illumination variation.

In terms of model complexity and inference speed, YOLO-Spectra-Net contains 4.2 M parameters, requires 11.8 GFLOPs, and achieves an average of 69 FPS. This computational cost is much lower than that of two-stage methods, while the average inference speed remains suitable for real-time processing. Considering accuracy and robustness together, the method provides a favorable balance between performance and deployment cost.

Training convergence also reflects optimization stability. As shown in Figure 6, YOLO-Spectra-Net increases mAP@0.5 rapidly during early training and stabilizes after approximately 70 epochs, with smooth curves and small fluctuations. Faster R-CNN and CenterNet converge more slowly and reach lower final performance. YOLOv8n and YOLOv12n converge quickly at the beginning, but their later-stage gains are limited and remain below those of the proposed method.

Grad-CAM visualizations further reveal differences in the feature response patterns of the compared models. As shown in Figure 7, the feature activation distributions of TOOD, Faster R-CNN, and CenterNet are relatively fragmented. Their high-response regions fail to cover the character targets completely, exhibit weak activation continuity across the character sequence, and partially spread to surrounding background structures. YOLOv8n and YOLOv12n focus their attention primarily on the character target regions, but their heatmaps still do not fully align with the character contours, with activation gaps appearing inside some characters. In contrast, YOLO-Spectra-Net (Ours) produces more uniform and continuous high responses over the character targets while suppressing ineffective activations in the background. This indicates that the proposed robustness enhancement mitigates feature activation noise under unstable modalities and improves feature discriminability under multiple interference conditions.

To supplement the qualitative Grad-CAM comparison, the localization quality of the heatmaps is quantitatively evaluated using the Pointing Game (PG) and Energy-Based Pointing Game (EBPG) metrics. The results on RailID-Dataset are reported in Table 6, and their overall comparison is visualized in Figure 8.

Taken together, the quantitative metrics, convergence curves, and visualizations demonstrate that YOLO-Spectra-Net achieves robust RGB–IR detection performance on the self-constructed dataset and provides stronger feature-response and training stability under complex interference.

### 4.8. Comparison Experiments on M3FD Dataset

On M3FD, single-modality methods are first compared under the same train/test split and input resolution, with the quantitative results summarized in Table 7. With RGB input only, YOLOv12n achieves 82.3% mAP@0.5 and 51.2% mAP@0.5:0.95; with infrared input only, the corresponding values are 78.9% and 50.3%. These results indicate that infrared sensing provides useful saliency in low-light scenes but may be limited in high-IoU localization, whereas RGB images preserve more spatial details when visibility is sufficient.

Fusion methods show clear performance differences. For SLBAF and U2Fusion [34], fused images are first generated using the authors’ released fusion pipelines and then evaluated with the same YOLOv12n detector training protocol; detector-level methods are trained directly with paired RGB–IR inputs. SLBAF obtains 44.3% mAP@0.5:0.95, which is lower than the RGB-only baseline, suggesting that image-level fusion may introduce redundancy or interference in this detection setting. DAMSDet [5] and U2Fusion reach 52.9% and 51.5% on the stricter metric, respectively, indicating more balanced use of multimodal information. TarDAL [4] obtains 50.8%, which is close to the best single-modality result but leaves room for improvement in complex scenes.

YOLO-Spectra-Net exhibits stable multimodal performance, reaching 82.7% mAP@0.5, which is tied with TarDAL and higher than the other compared methods on this metric. On mAP@0.5:0.95, it achieves 52.3%, outperforming TarDAL, U2Fusion, and SLBAF, while remaining slightly lower than DAMSDet. It also achieves an average of 66 FPS with 4.8 M parameters and 13.6 GFLOPs, which is faster than the other detector-level multimodal methods listed in the table. This comparison indicates that the main advantage on M3FD lies in stable high-confidence detection, competitive strict-IoU localization, and practical inference efficiency rather than a uniform lead on every metric. The proposed method preserves recall while improving localization robustness over most baselines, which is beneficial for small targets and cluttered backgrounds.

Grad-CAM visualizations further reveal response stability differences. As shown in Figure 9, RGB-only detection can generate strong target responses but remains unstable in low-light regions. SLBAF and some fusion methods produce diffused responses with attention distributed over non-target regions, indicating insufficient suppression of redundant fusion responses. DAMSDet, U2Fusion, and TarDAL improve target activation but still exhibit blurred boundaries or discontinuous local responses.

The quantitative heatmap localization results on M3FD are presented in Table 8. PG and EBPG provide complementary measurements of whether the strongest and overall heatmap responses are concentrated within target regions, and their model-wise comparison is shown in Figure 10.

YOLO-Spectra-Net generates more concentrated and continuous high responses on targets while reducing background activation. Vehicle contours and key structures are more completely covered, suggesting that the model effectively integrates RGB detail with infrared saliency while suppressing conflicting responses. This observation is consistent with the intended roles of modality gating, complementary fusion, and conflict-aware suppression.

Combining quantitative metrics and visualizations, YOLO-Spectra-Net improves detection reliability on M3FD and produces more stable target-focused responses under adverse multimodal sensing conditions. These results indicate that explicitly modeling modality reliability and suppressing cross-modal inconsistency are important for maintaining stable perception in practical infrared-visible systems.

### 4.9. Comparison Experiments on FLIR Dataset

Single-modality and multimodal methods show clear differences on FLIR, as summarized in Table 9. Using YOLOv12n as the baseline, RGB-only detection achieves 57.9% mAP@0.5 and 26.6% mAP@0.5:0.95, whereas infrared-only detection reaches 74.7% and 37.1%, respectively. This indicates that infrared sensing provides more stable responses in low-light or night-time scenes, while RGB information alone is relatively limited on this dataset.

Among multimodal methods, fusion strategies improve performance to different extents. GAFF [35] obtains 72.9% mAP@0.5 and 37.5% mAP@0.5:0.95, outperforming RGB-only detection but still remaining below the infrared-only baseline, suggesting that multimodal information is not fully exploited. YOLO-MS [8], MRD-YOLO [36], CFT [6], and CSAA [37] further improve the stricter metrics; CSAA reaches 79.2% mAP@0.5 and 41.4% mAP@0.5:0.95, demonstrating that effective fusion can maintain performance across IoU thresholds.

The proposed method achieves competitive multimodal performance, reaching 79.1% mAP@0.5, which is 0.1 percentage points lower than CSAA, and 41.5% mAP@0.5:0.95, which is 0.1 percentage points higher than CSAA. It achieves an average of 71 FPS with 4.8M parameters and 13.6 GFLOPs, exceeding the average inference speeds of the other compared multimodal methods except GAFF. Although the absolute accuracy difference from the strongest baseline is marginal, SNYOLO combines competitive localization with a relatively compact multimodal structure and practical inference efficiency. The strict-IoU result suggests stable localization and boundary regression, while the complexity metrics support practical deployment.

Grad-CAM results further reveal differences in feature responses. As shown in Figure 11, RGB-only detection produces weak activation in low-light regions, and some targets are insufficiently activated. Certain multimodal methods still show diffused or missing responses after fusion, indicating residual redundancy or interference.

The corresponding heatmap localization results on FLIR are summarized in Table 10. These results provide an additional quantitative comparison of target-focused responses under visible and infrared sensing conditions, with the model-wise PG and EBPG comparison further illustrated in Figure 12.

In comparison, the proposed method produces more concentrated and continuous high responses on target regions, covering vehicle contours and key structures while reducing background activation. This indicates that, during RGB–IR fusion, the model adaptively selects more discriminative modality information at different locations and suppresses inter-modality interference. Through modality gating, complementary fusion, and conflict-aware suppression, SNYOLO captures multimodal information more effectively at the feature level and improves perception reliability under complex lighting.

Considering metrics, complexity, and visualizations together, SNYOLO matches the strongest compared methods on FLIR in accuracy while requiring fewer parameters and FLOPs. It also improves response stability under complex lighting, further verifying that reliability-aware multimodal fusion can stabilize feature behavior under modality degradation and environmental variation.

The joint relationship among mAP@0.5, average inference speed, and model size is summarized in Figure 13. The comparison indicates that SNYOLO provides a relatively lightweight multimodal solution with practical average inference speed and competitive mAP@0.5. This claim is made relative to the compared multimodal detectors; some single-modality baselines have fewer parameters or lower FLOPs, but they do not perform the same RGB–IR fusion task.

### 4.10. Ablation Experiment Analysis

To clarify the contribution of each mechanism to robustness, ablation experiments are conducted on RailID-Dataset, which provides paired RGB–IR inputs. Starting from the RGB-only YOLOv12n baseline, paired multimodal fusion, CMGF-based adaptive fusion, multi-scale consistency modeling, and conflict-aware suppression are added progressively and evaluated under the same dataset and training setup. The results are reported in Table 11.

Without any added modules, the baseline uses only visible images for detection and obtains 82.3% mAP@0.5 and 84.1% Precision. Without multimodal fusion and reliability modeling, the detector is more sensitive to illumination variation and complex backgrounds, leading to less stable predictions and a higher risk of false or missed detections.

When paired RGB + IR fusion is introduced through direct feature addition, mAP@0.5 increases to 82.4% and Precision to 84.5%. This modest gain reflects multimodal complementarity: visible images provide texture and structure, whereas infrared images provide more stable responses under weak illumination or occlusion. The limited improvement also indicates that simple fusion does not sufficiently address modality inconsistency.

Adding CMGF on top of fusion increases mAP@0.5 to 82.5% and Precision to 85.0%. This indicates that cross-modal global fusion improves robustness by modeling spatial–channel importance and regulating cross-modal information flow before feature aggregation. When modality quality varies across scenes, adaptive gating helps stabilize feature interaction and reduce error propagation.

Adding multi-scale consistency modeling further increases mAP@0.5 to 82.6% and Precision to 85.2%. Without this component, features from different scales can remain inconsistent, reducing robustness for targets with varying sizes. Although the gain is moderate, the result suggests improved prediction consistency under complex backgrounds by aligning deep semantic cues with shallow spatial details.

Incorporating conflict-aware suppression yields the best result, with 82.7% mAP@0.5 and 85.6% Precision. Without this component, ambiguous activations caused by modality disagreement can propagate and destabilize fused features. CASS suppresses unreliable responses, maintains high recall while reducing false detections, and provides more stable improvements under dense targets or heavy occlusion.

To compare with matched simple alternatives, direct addition and concatenation fusion are also tested under the same backbone. Direct addition corresponds to Sequence 2 in Table 11; concatenation followed by a 1×1 convolution reaches 82.4% mAP@0.5 and 84.7% Precision. Both results are lower than those of the CMGF-based design, indicating that the improvement is not merely caused by adding a second modality or increasing channel capacity.

Controlled degradation experiments further validate the reliability mechanism. When synthetic fog is applied to RGB inputs on RailID-Dataset, the average RGB gate decreases while the infrared gate increases, and the detector maintains more stable target responses than direct fusion. Weight-map visualizations show higher RGB weights in texture-rich daytime regions and higher infrared weights in dark or low-contrast target regions. Grad-CAM visualizations in Figure 14 also support these quantitative observations.

### 4.11. Resolution Sensitivity Analysis on M3FD

To evaluate the scalability of SNYOLO to higher-resolution inputs, experiments are conducted on the M3FD dataset using the same paired RGB–IR data split and model configuration. The input resolution is varied from 512×512 to 1024×1024, while the remaining training and evaluation settings are kept unchanged. The results are summarized in Table 12.

The relationship between detection accuracy and average inference speed under different input resolutions is visualized in Figure 15. The two curves show the changes in mAP@0.5 and mAP@0.5:0.95 with respect to average FPS, while the point labels indicate the corresponding input resolutions.

As the input resolution increases, the detection accuracy generally improves, especially at 1024×1024, where mAP@0.5 and mAP@0.5:0.95 reach 84.3% and 53.3%, respectively. Compared with the 640×640 setting, the 1024×1024 setting improves mAP@0.5 by 1.6 percentage points and mAP@0.5:0.95 by 1.0 percentage point, indicating that higher-resolution inputs can provide additional spatial information for detection and localization. The number of parameters remains unchanged at 4.8M because the network architecture is not modified.

The improvement in accuracy is accompanied by increased computational cost and reduced average inference speed. GFLOPs increase from 13.6 at 640×640 to 35.8 at 1024×1024, while average FPS decreases from 66 to 31. The 640×640 setting therefore provides a more balanced trade-off between accuracy and real-time efficiency, whereas the 1024×1024 setting is more suitable for applications that prioritize accuracy over speed. These results demonstrate that SNYOLO can be extended to higher-resolution RGB–IR inputs without changing its overall architecture, although the associated memory and computational requirements should be considered in practical deployment.

## 5. Conclusions and Future Work

### 5.1. Conclusions

To address illumination variation, modality degradation, and background interference under adverse sensing conditions, this work presents YOLO-Spectra-Net (SNYOLO), a reliability-aware RGB–infrared fusion framework for robust object detection. Built on a YOLO-style architecture, SNYOLO integrates the CMGF module with spatial–channel attention, cross-modal global interaction, adaptive gating, multi-scale perception modeling, and conflict-aware spatial suppression. The framework exploits the complementarity between visible and infrared sensing while maintaining efficient inference.

Experiments on the self-collected paired RGB–IR RailID-Dataset and two public paired multimodal datasets, M3FD and FLIR, show that the proposed design improves detection reliability in challenging environments, with smoother convergence and more stable target-focused responses under illumination changes and complex interference. RailID verifies RGB–IR fusion robustness under controlled degradation, whereas M3FD and FLIR evaluate generalization on paired public data. These results support the applicability of reliability-aware multimodal fusion to safety-critical scenarios such as railway monitoring and surveillance, where robust perception is required under non-ideal sensing conditions.

### 5.2. Future Work

Although the proposed method shows stable gains across datasets, several directions remain for future research. The current framework models static images and does not exploit temporal information from consecutive frames in real-world surveillance videos. Future work will investigate lightweight temporal modeling or cross-frame feature constraints to improve robustness in continuous operation.

In addition, the self-collected dataset remains limited in scale and scene diversity. Future work will expand acquisition environments and sensor configurations to enrich the data distribution and improve model generalization [38]. For deployment, model compression and acceleration will be considered to further optimize the network for edge devices.

## Figures and Tables

**Figure 1:**
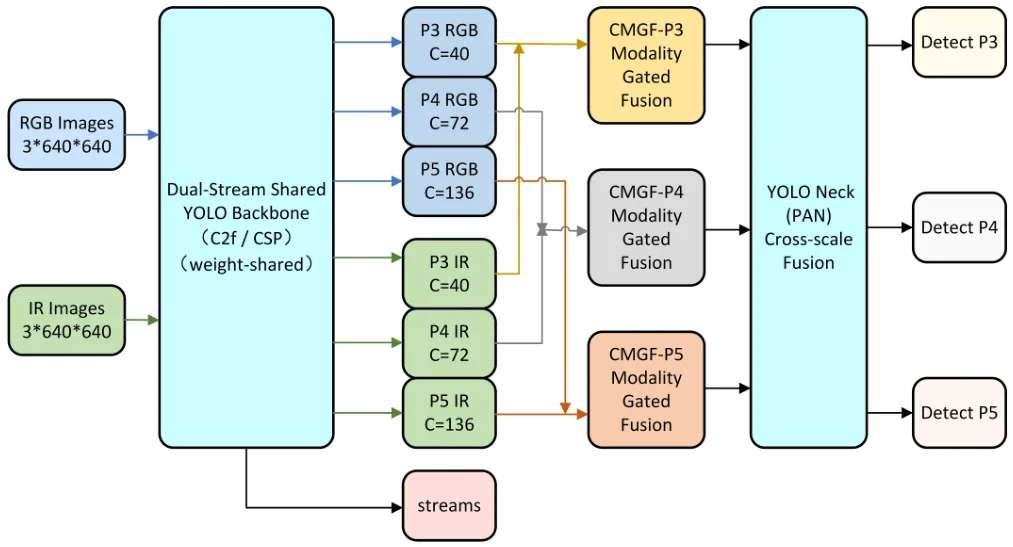
Overall framework of YOLO-Spectra-Net (SNYOLO).

**Figure 2:**
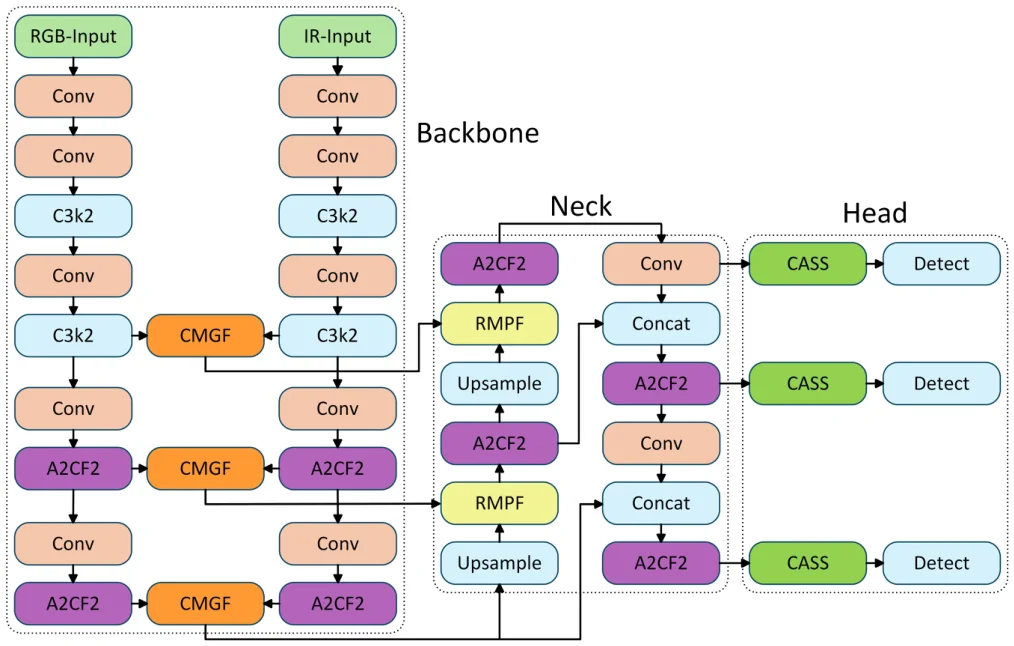
Detailed network structure of YOLO-Spectra-Net (SNYOLO), including the locations of CMGF, RMPF, and CASS.

**Figure 3:**
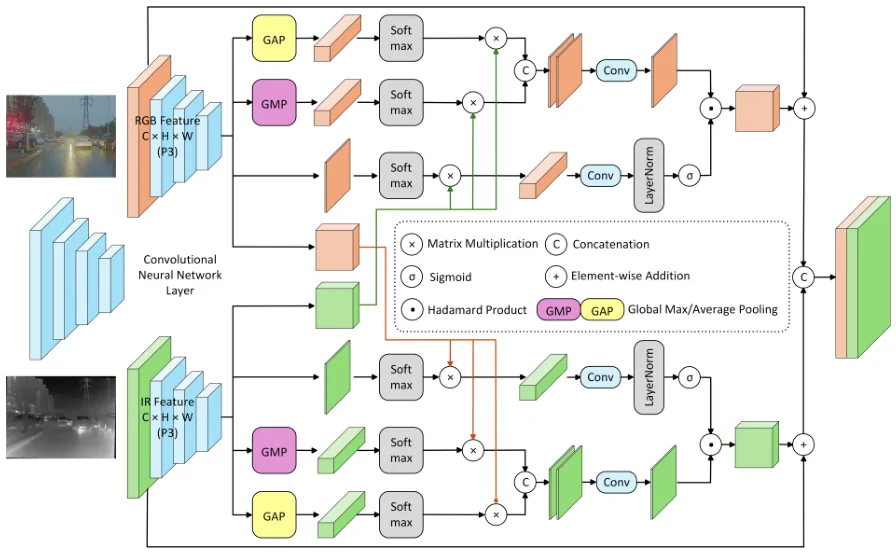
Structure of the Cross-Modal Global Fusion (CMGF) module. CMGF combines spatial–channel attention, cross-modal global interaction, and adaptive gated fusion to generate the fused RGB–infrared feature.

**Figure 4:**
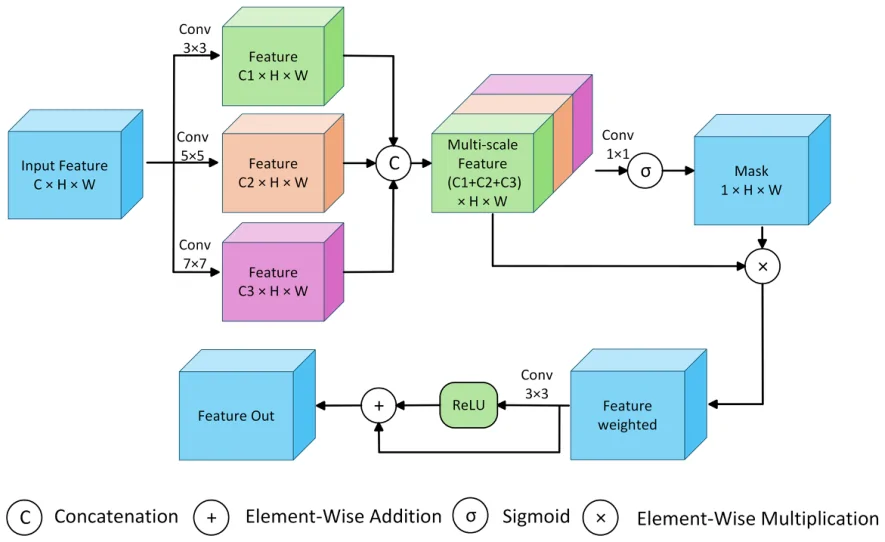
Structure of the RMPF module.

**Figure 5:**
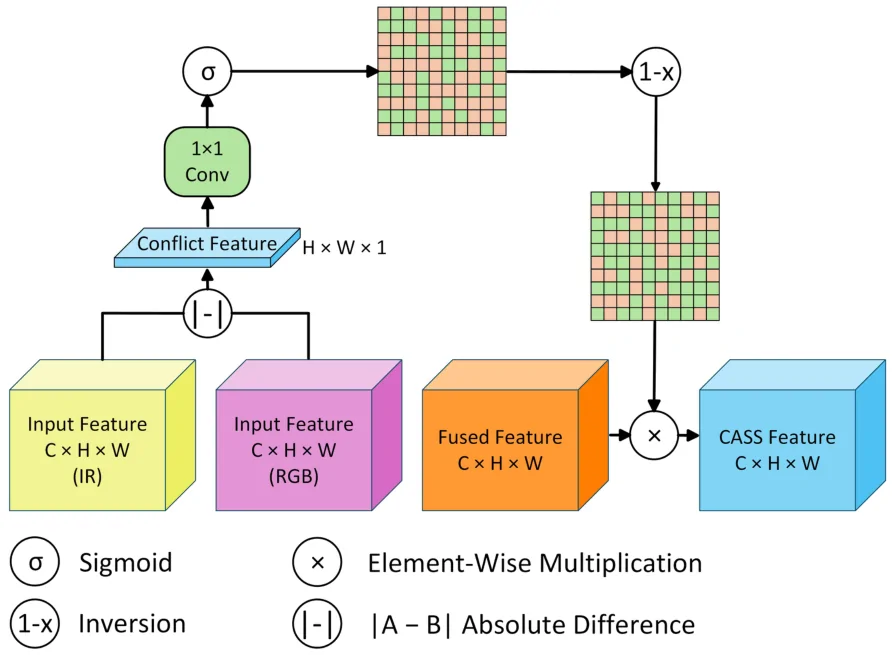
Structure of the CASS module.

**Figure 6:**
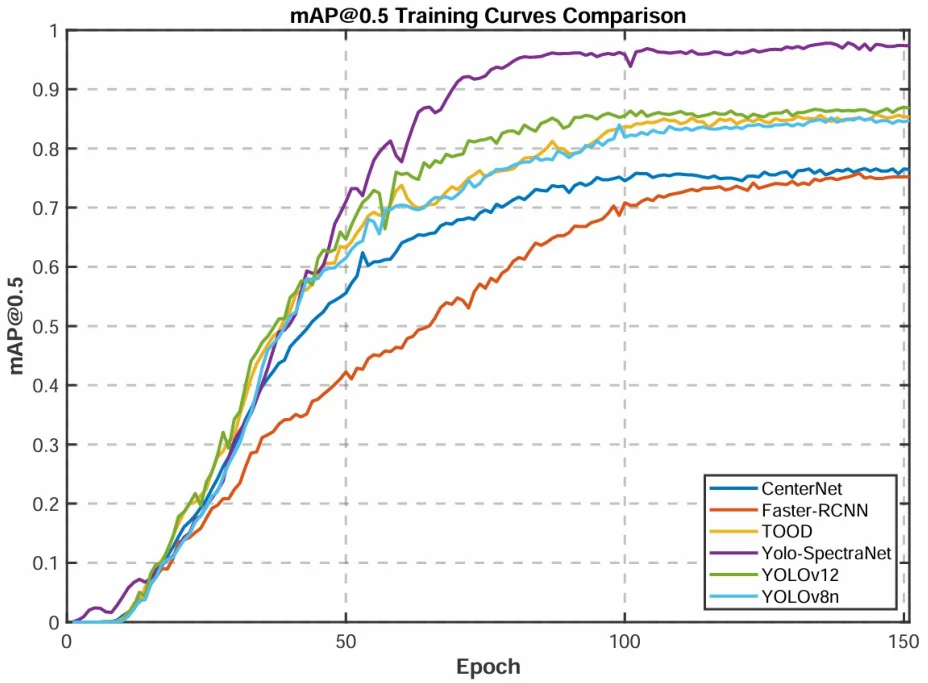
Training mAP@0.5 convergence curves on RailID-Dataset.

**Figure 7:**
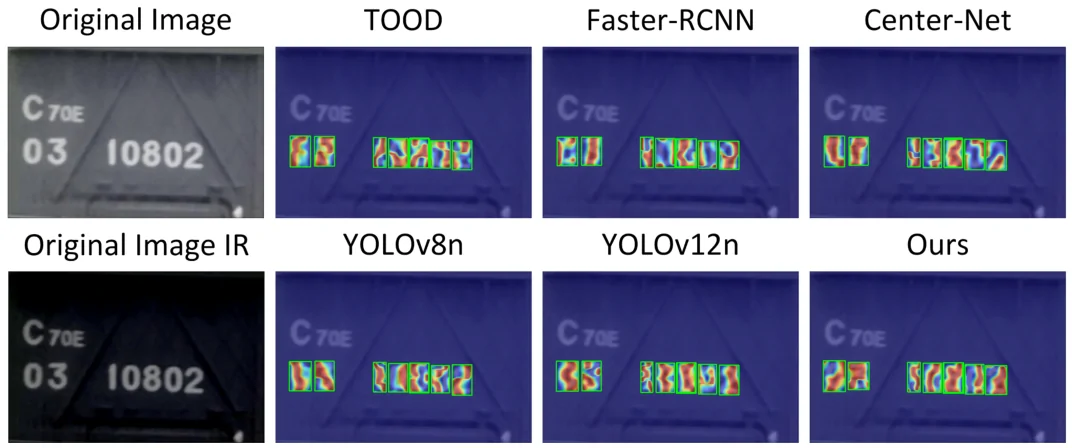
Grad-CAM visualization on RailID-Dataset.

**Figure 8:**
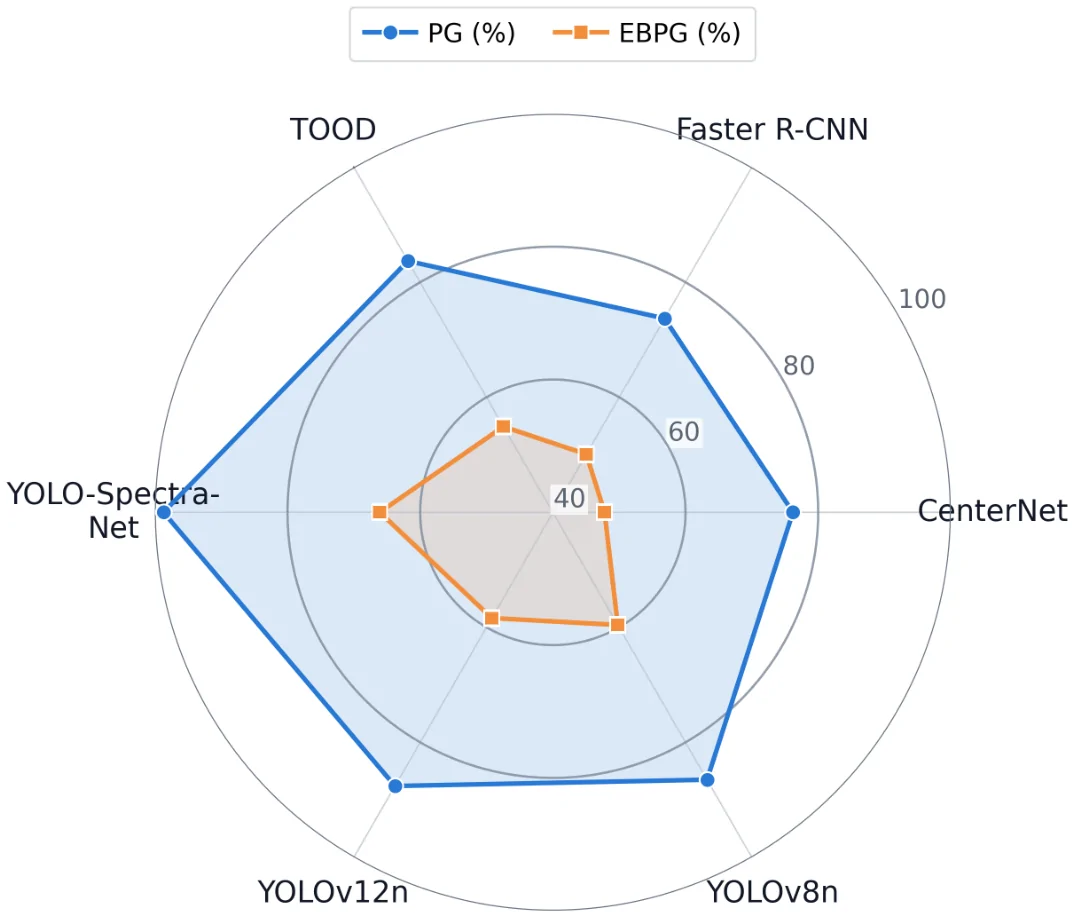
Radar-chart comparison of PG and EBPG heatmap localization results on RailID-Dataset. The radial axes correspond to the compared models.

**Figure 9:**
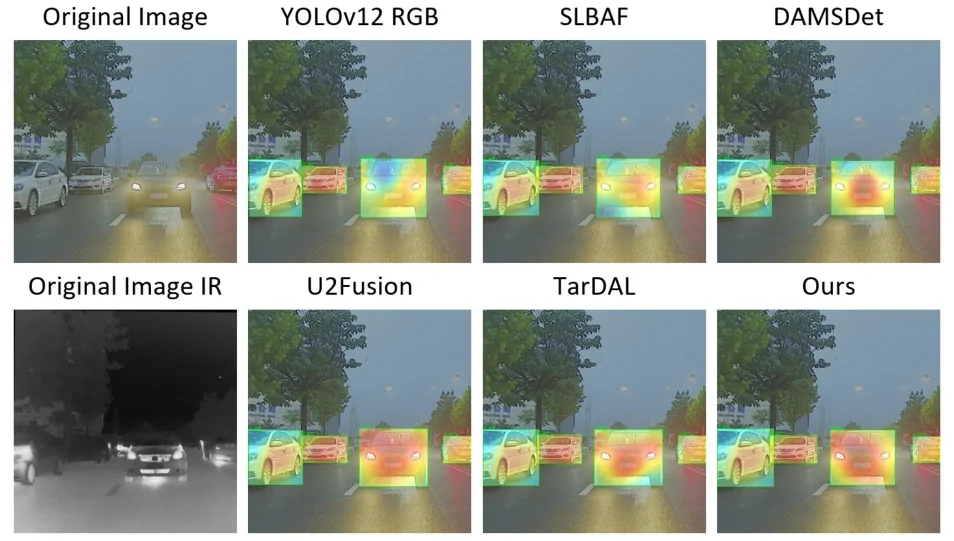
Grad-CAM visualization on M3FD.

**Figure 10:**
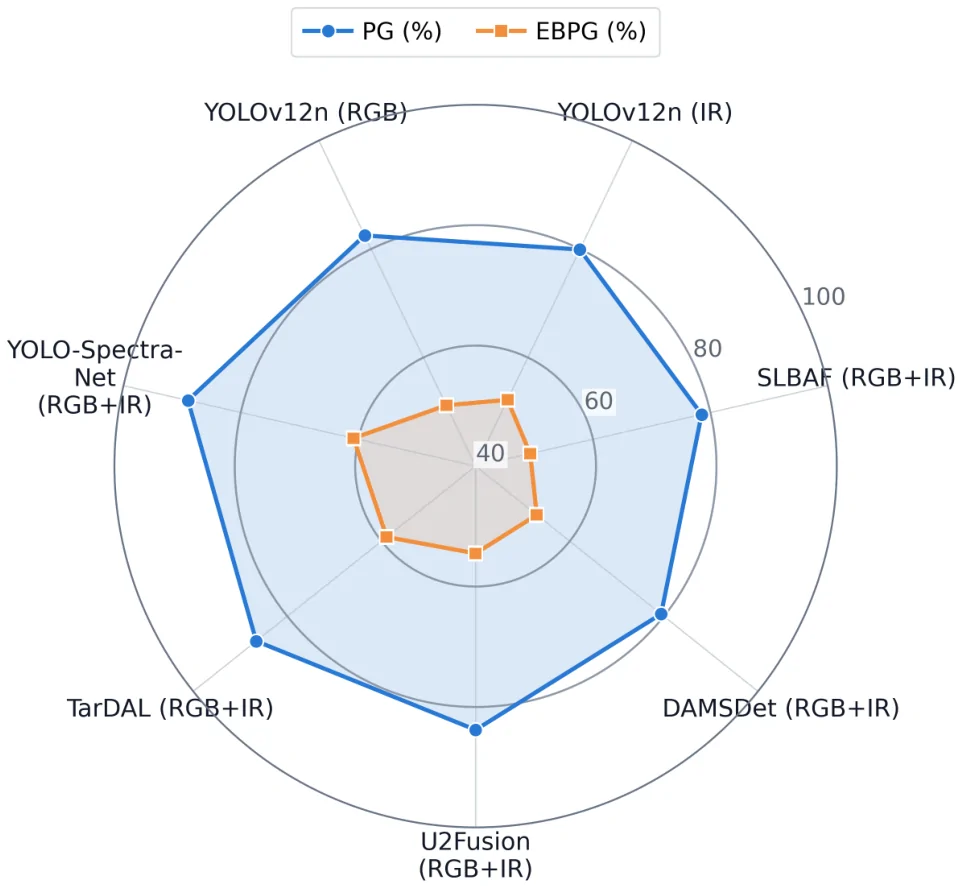
Radar-chart comparison of PG and EBPG heatmap localization results on the M3FD dataset. The radial axes correspond to the compared models.

**Figure 11:**
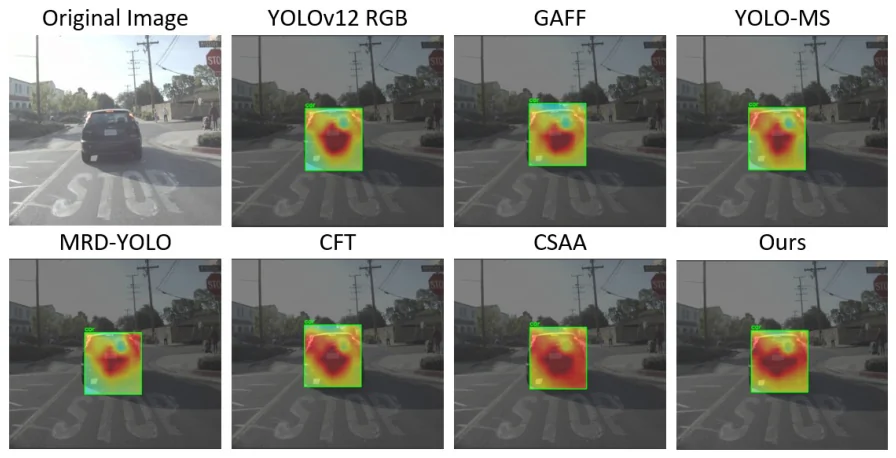
Grad-CAM visualization on FLIR.

**Figure 12:**
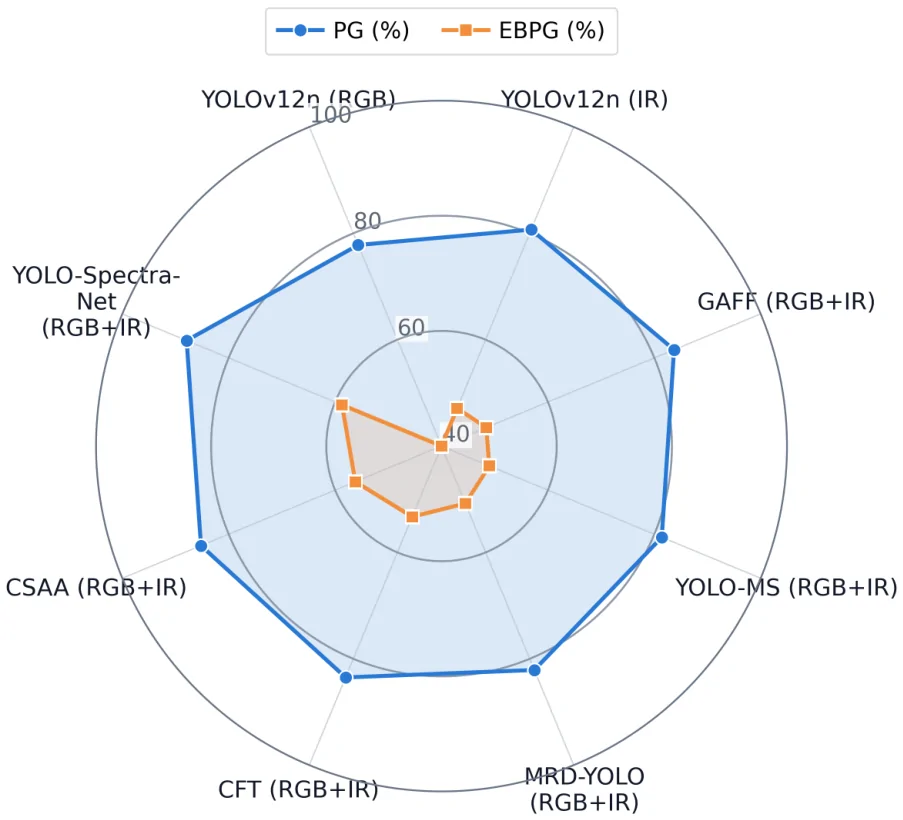
Radar-chart comparison of PG and EBPG heatmap localization results on the FLIR dataset. The radial axes correspond to the compared models.

**Figure 13:**
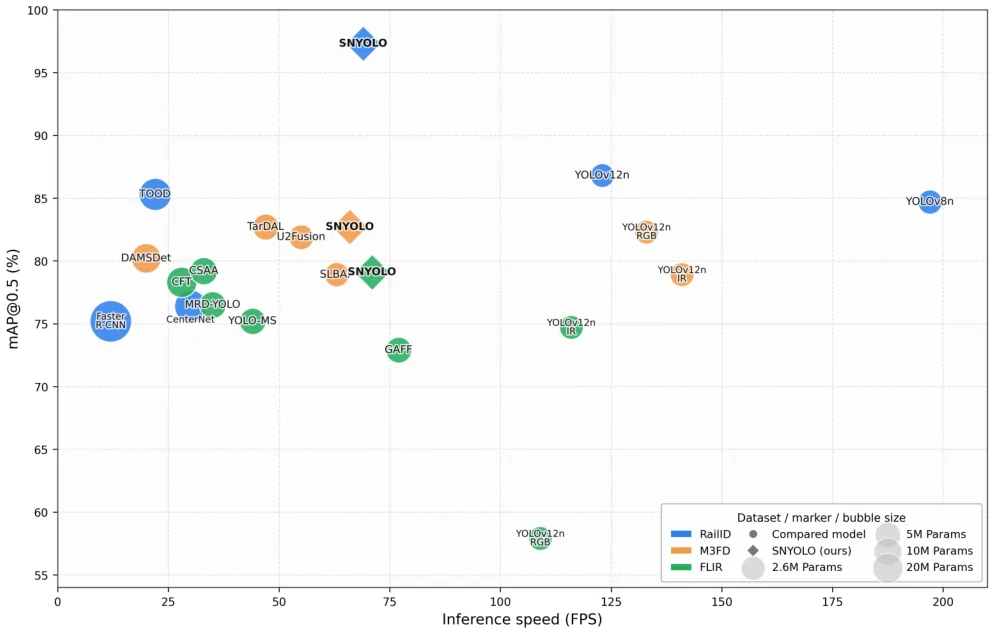
Joint comparison of mAP@0.5, average inference speed, and parameter count across the RailID, M3FD, and FLIR datasets. Bubble size represents the number of parameters, and diamond markers denote SNYOLO.

**Figure 14:**
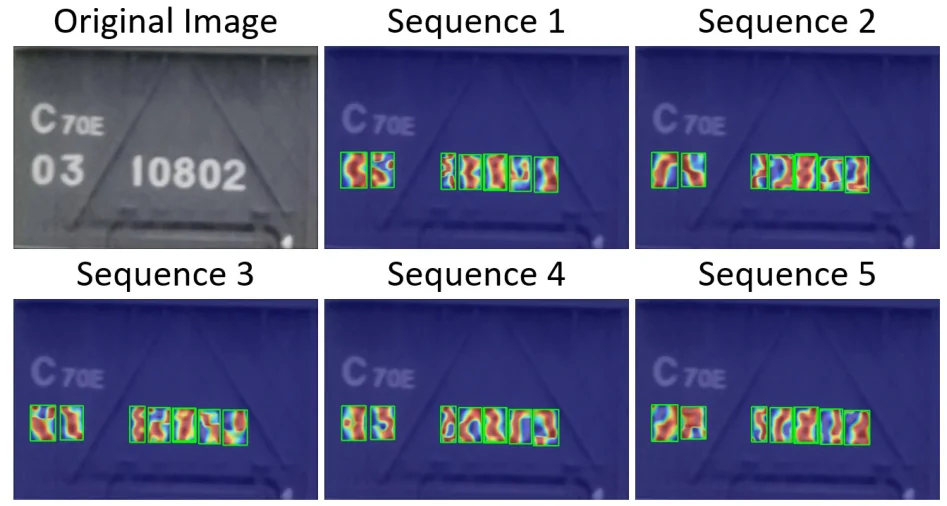
Grad-CAM visualization of the ablation study on RailID-Dataset.

**Figure 15:**
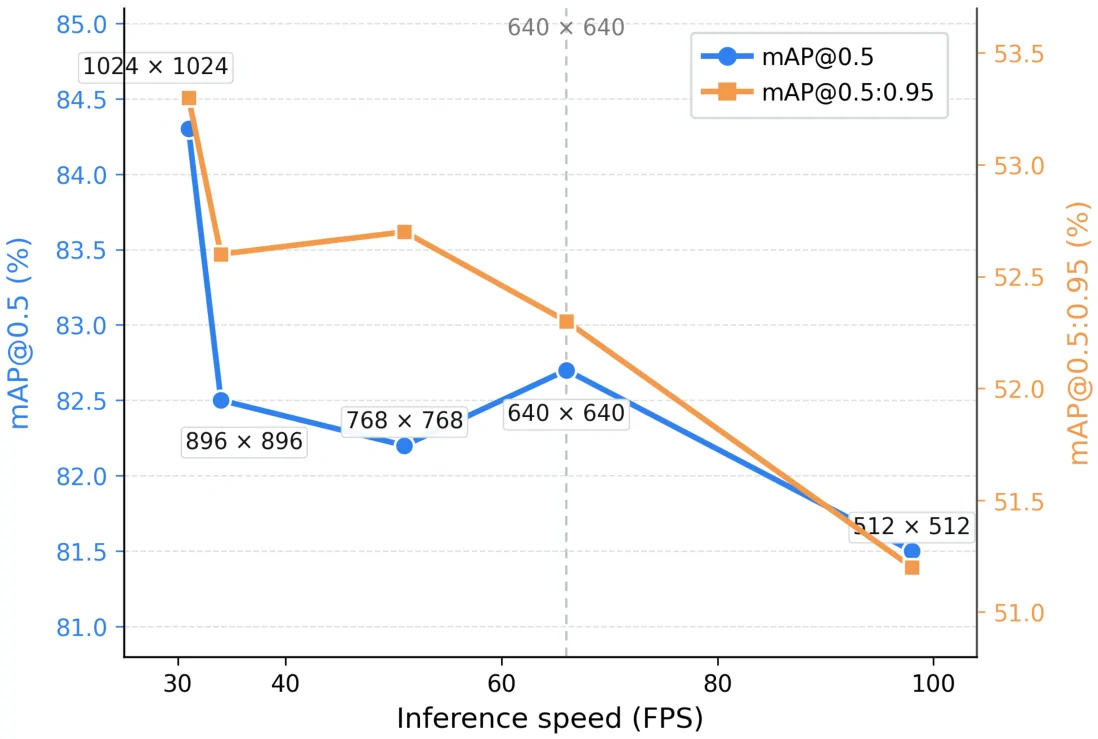
Accuracy–speed relationship of SNYOLO under different input resolutions on the M3FD dataset. The horizontal axis represents average inference speed (FPS), and the two curves represent mAP@0.5 and mAP@0.5:0.95, respectively. Point labels indicate the input resolutions in pixels.

**Table 1:** Representative single-modality object detection methods.

Model	Paradigm	Strengths	Limitations
R-CNN	Two-stage	High localization accuracy; early deep-learning detector	Slow inference; complex pipeline
Faster R-CNN	Two-stage	End-to-end training; stable performance	Limited real-time capability; high cost
SSD	One-stage	Simple structure; fast inference	Limited small-object performance
YOLOv12	One-stage	Balanced accuracy and speed; engineering-friendly	Robustness in complex scenes can be improved
DETR	Transformer-based	Strong global modeling; reduced hand-crafted priors	Slow convergence; high compute demand

**Table 2:** Attention mechanisms for multimodal fusion.

Type	Main Function	Limitations
Channel attention [12]	Enhances discriminative channels; improves selectivity	Ignores spatial distribution; weak local modeling
Spatial attention [13]	Emphasizes key regions; improves localization	Insufficient adaptation to scale variation
Cross-modality attention [3]	Models modality correlation; improves complementarity	Often symmetric; ignores modality differences
Multi-scale attention [14]	Fuses multi-receptive-field cues; improves scale robustness	Complex structure; higher computational cost
Global attention [11]	Models long-range dependencies; enhances global semantics	Insufficient fine-grained local focus
Region-aware attention [15]	Assigns region-wise weights	Often lacks explicit conflict suppression

**Table 3:** Hardware and software environment.

Item	Configuration
Operating system	Ubuntu 22.04 LTS
Deep learning framework	PyTorch 2.2
GPU	NVIDIA RTX 4090 (24 GB)
CPU	Intel Core i9-13900K
CUDA/cuDNN	12.2/8.9
Language	Python 3.10
Data processing	OpenCV 4.12.0, NumPy 2.2.6, Pandas 2.3.2

**Table 4:** Training hyperparameters used in all experiments.

Hyperparameter	Value
Input resolution	640×640
Batch size	16
Data workers	4
Total training epochs	150
Optimizer	SGD
Initial learning rate	0.01
Momentum	0.937
Weight decay	0.0005
Mosaic augmentation	Disabled during the last 10 epochs

**Table 5:** Results on the self-collected RailID-Dataset. “Pre-fused (single)” denotes one image generated by combining the corresponding RGB and infrared images before detection, whereas “Original pair (dual)” denotes the two separate aligned RGB and infrared inputs used by SNYOLO.

Model	Input Format (Single/Dual)	mAP@0.5 (%)	mAP@0.5:0.95 (%)	Precision (%)	Recall (%)	Params (M)	GFLOPs	Average FPS (Frames/s)
TOOD [31]	Pre-fused (single)	85.3	60.1	85.0	80.0	32.0	68.4	22
Faster R-CNN [16]	Pre-fused (single)	75.2	50.1	76.0	68.0	137.1	180.2	12
CenterNet [32]	Pre-fused (single)	76.4	51.3	74.0	70.0	32.7	72.6	30
YOLOv8n [33]	Pre-fused (single)	84.7	58.9	82.0	78.0	3.1	8.7	197
YOLOv12n [9]	Pre-fused (single)	86.8	60.5	84.0	79.0	2.6	6.5	123
YOLO-Spectra-Net (Ours)	Original pair (dual)	97.3	68.2	96.0	91.0	4.2	11.8	69

**Table 6:** Heatmap localization results on the self-collected RailID-Dataset. “Pre-fused (single)” denotes one image generated by combining the corresponding RGB and infrared images before detection, whereas “Original pair (dual)” denotes the two separate aligned RGB and infrared inputs used by SNYOLO.

Model	Input Format (Single/Dual)	PG (%)	EBPG (%)
TOOD [31]	Pre-fused (single)	83.7	54.9
Faster R-CNN [16]	Pre-fused (single)	73.7	50.1
CenterNet [32]	Pre-fused (single)	76.2	47.8
YOLOv8n [33]	Pre-fused (single)	86.5	59.6
YOLOv12n [9]	Pre-fused (single)	87.6	58.4
YOLO-Spectra-Net (Ours)	Original pair (dual)	98.7	66.1

**Table 7:** Results on the M3FD dataset.

Method	Data	mAP@0.5 (%)	mAP@0.5:0.95 (%)	Params (M)	GFLOPs	Average FPS (Frames/s)
YOLOv12n	RGB	82.3	51.2	2.6	6.5	133
YOLOv12n	IR	78.9	50.3	2.6	6.5	141
SLBAF	RGB + IR	78.9	44.3	3.4	15.2	63
DAMSDet [5]	RGB + IR	80.2	52.9	18.6	46.8	20
U2Fusion [34]	RGB + IR	81.9	51.5	4.1	18.5	55
TarDAL [4]	RGB + IR	82.7	50.8	6.8	24.7	47
YOLO-Spectra-Net (Ours)	RGB + IR	82.7	52.3	4.8	13.6	66

**Table 8:** Heatmap localization results on the M3FD dataset.

Method	Data	PG (%)	EBPG (%)
YOLOv12n	RGB	82.5	51.2
YOLOv12n	IR	79.9	52.2
SLBAF	RGB + IR	78.5	49.3
DAMSDet [5]	RGB + IR	79.4	53.0
U2Fusion [34]	RGB + IR	83.8	54.5
TarDAL [4]	RGB + IR	86.6	58.9
YOLO-Spectra-Net (Ours)	RGB + IR	89.0	60.8

**Table 9:** Results on the FLIR dataset.

Method	Data	mAP@0.5 (%)	mAP@0.5:0.95 (%)	Params (M)	GFLOPs	Average FPS (Frames/s)
YOLOv12n	RGB	57.9	26.6	2.6	6.5	109
YOLOv12n	IR	74.7	37.1	2.6	6.5	116
GAFF [35]	RGB + IR	72.9	37.5	5.9	21.3	77
YOLO-MS [8]	RGB + IR	75.2	38.3	7.2	25.1	44
MRD-YOLO [36]	RGB + IR	76.5	40.9	8.4	28.6	35
CFT [6]	RGB + IR	78.3	40.2	21.5	53.4	28
CSAA [37]	RGB + IR	79.2	41.4	9.8	31.7	33
YOLO-Spectra-Net (Ours)	RGB + IR	79.1	41.5	4.8	13.6	71

**Table 10:** Heatmap localization results on the FLIR dataset.

Method	Data	PG (%)	EBPG (%)
YOLOv12n	RGB	77.8	39.8
YOLOv12n	IR	80.7	47.1
GAFF [35]	RGB + IR	83.7	48.4
YOLO-MS [8]	RGB + IR	81.4	49.0
MRD-YOLO [36]	RGB + IR	82.1	50.8
CFT [6]	RGB + IR	83.5	53.3
CSAA [37]	RGB + IR	85.2	56.2
YOLO-Spectra-Net (Ours)	RGB + IR	87.9	58.7

**Table 11:** Ablation study on paired RGB–IR RailID-Dataset data. Here, ✔ indicates that the corresponding component is enabled, whereas × indicates that it is disabled.

Sequence	RGB + IR	CMGF	RMPF	CASS	mAP@0.5 (%)	Precision (%)
1	×	×	×	×	82.3	84.1
2	✔	×	×	×	82.4	84.5
3	✔	✔	×	×	82.5	85.0
4	✔	✔	✔	×	82.6	85.2
5	✔	✔	✔	✔	82.7	85.6

**Table 12:** Performance of SNYOLO under different input resolutions on the M3FD dataset.

Resolution	mAP@0.5 (%)	mAP@0.5:0.95 (%)	Params (M)	GFLOPs	Average FPS (Frames/s)
512×512	81.5	51.2	4.8	9.1	98
640×640	82.7	52.3	4.8	13.6	66
768×768	82.2	52.7	4.8	17.9	51
896×896	82.5	52.6	4.8	23.4	34
1024×1024	84.3	53.3	4.8	35.8	31

## Data Availability

The M3FD and FLIR datasets analyzed in this study are publicly available from their respective dataset providers. The self-collected RailID-Dataset is not publicly available at present; the data supporting the findings of this study are available from the corresponding author upon reasonable request.
